# Impact of Silica Nanoparticles on Mechanical Properties and Self-Healing Performance of PVA Hydrogels

**DOI:** 10.3390/polym17212883

**Published:** 2025-10-29

**Authors:** Moustapha Mohamed Mahamoud, Yutaka Kuwahara, Hirotaka Ihara, Makoto Takafuji

**Affiliations:** 1 Faculty of Advanced Science and Technology, Kumamoto University, 2-39-1 Kurokami, Chuo-ku, Kumamoto 860-8555, Japan; harroun92@gmail.com (M.M.M.);; 2 International Research Organization for Advanced Science and Technology (IROAST), Kumamoto University, 2-39-1 Kurokami, Chuo-ku, Kumamoto 860-8555, Japan

**Keywords:** hydrogels, silica nanoparticles, polyvinyl alcohol, self-healing

## Abstract

Hydrogels are three-dimensional polymeric networks capable of retaining large amounts of water. Polyvinyl alcohol (PVA)-based hydrogels exhibit autonomous self-healing through reversible physical interactions within the hydrogel matrix, including hydrogen bonding, crystallite formation, and dynamic crosslinking. However, their long self-healing times and low strength limit practical application. Herein, we propose an effective strategy to simultaneously achieve excellent self-repairing and high mechanical strength. The tensile strength of uncut PVA hydrogel was 1.21 MPa; after cutting and rejoining for 12 h at room temperature (RT), it recovered 94% of the original uncut strength. To accelerate self-healing, hydrogels were treated at 40, 50, and 60 °C for 20, 40, and 60 min. Under optimal conditions (60 °C for 60 min), 96% recovery was achieved. Mechanical properties were further improved by silica (Si) nanoparticles of various sizes (~12, ~85, and ~200 nm). Si-loaded hydrogels, particularly ~12 nm, demonstrated increased mechanical properties, reaching a tensile strength of 1.45 MPa and a self-healing recovery of 95% of the uncut hydrogel strength. Ultra-small (~12 nm) Si nanoparticles enhanced the overall mechanical properties by acting as an efficient nucleating agent and did not hinder the existing self-healing mechanism. The developed strategy will pave the way for novel techniques in hydrogel research and will advance applications such as soft robotics and wound dressing.

## 1. Introduction

Versatile hydrogels with high mechanical strength and intrinsic self-healing ability have attracted considerable attention in recent years owing to their promising potential in advanced applications, including flexible and durable artificial skin, controlled drug delivery systems, and next-generation energy storage devices. The synergy between robustness and autonomous repairability not only enhances the longevity and reliability of the hydrogel material but also broadens its applicability [1,2]. However, achieving both high mechanical strength and efficient self-healing remains a major challenge. The self-healing mechanism of hydrogels primarily arises from dynamic interactions or hydrogen bonding mechanisms [3,4,5].

Extensive studies have sought to enhance the mechanical strength of hydrogels by integrating Si nanoparticles, constructing dual-network frameworks, and introducing supramolecular interactions [6,7,8]. Lee et al. showed that Si nanoparticles act as effective nucleating agents at 0.5 wt%, with 22 nm particles providing optimal spacing for crystal growth and yielding ~20% higher crystallinity than pristine PVA, while smaller and larger sizes were less effective [9]. Chen et al. studied the crystallization of thermoplastic polyester elastomers (TPEE) with 20 nm and 50 nm Si (pristine and surface-treated) and found that all acted as strong nucleators, raising crystallization temperature by 20–30 °C without altering the crystal structure. Surface-treated 20 nm Si showed the highest activity, while 20 nm particles increased viscosity and slowed crystal growth; in contrast, 50 nm Si, though weaker as a nucleator, enabled the fastest crystallization due to lower viscosity effects [10]. Annaka et al. developed an injectable hydrogel composite for intraocular applications, consisting of hydrophobically modified polyethylene glycol (PEG) combined with hydrophilized Si nanoparticles (2–5 nm) [11]. Yang et al. introduced Si nanoparticles (30 nm) as crosslinking nodes within poly(acrylamide)-based hydrogels, leading to a remarkable enhancement of mechanical properties [12]. In addition to improving mechanical strength by Si nanoparticles, researchers investigated the self-healing properties. Zhang et al. showed that freeze–thaw PVA hydrogels autonomously self-heal at room temperature, restoring ~40% of their tensile strength within 1 h and reaching ~72% after 48 h through hydrogen bonding between polymer chains [13]. Zengin et al. developed thiol-functionalized mesoporous Si nanoparticles (MSNs) that act as dynamic crosslinkers with PEG hydrogels via thiol–disulfide exchange reactions. The strategy produced nanocomposites with significantly enhanced mechanical strength (storage modulus up to 32 ± 5 kPa vs. 1.3 ± 0.3 kPa for PEG alone) and rapid self-healing ability. In contrast, non-modified MSNs provided limited reinforcement (3.4 ± 0.7 kPa) [14]. Huang et al. developed a high-strength, self-healing nano-silica hydrogel exhibiting rapid autonomous repair (within 15 s), excellent stretchability up to 1200%, and anisotropic conductivity, making it highly promising for applications in nanoelectronics and electrochemical devices [15]. Zheng et al. fabricated mechanically robust and self-healable supramolecular hydrogels by employing poly(2-dimethylaminoethyl-methacrylate)-grafted Si nanoparticles (~100 nm) as multifunctional macro-crosslinkers within a poly (acrylic acid) network, achieving high tensile strength, efficient autonomous self-healing at RT, and water-induced shape-memory behaviour [16]. It is well reported that the mechanical and physical behaviour of semicrystalline polymers is dictated by their molecular architecture, which is largely determined by the crystallization process. Therefore, controlling polymer crystallization provides a viable approach to tailor and design the ultimate hydrogel material properties.

In this work, we reported the development of a novel hydrophilic hydrogel system that exhibits multiple advanced functionalities, most notably the combination of excellent self-healing capability and high mechanical strength. The design of this material addresses a central challenge in hydrogel engineering, namely the imbalance between dynamic reversibility for self-repair and structural robustness for mechanical durability. To overcome this limitation, we introduced Si nanoparticles (mesoporous and microporous) due to their adjustable size, uniform nanostructure, and aqueous stability. Si nanoparticles have become important inorganic fillers due to their hydrophilic surfaces and high surface area, which promote strong interfacial bonding and effective load transfer within hydrophilic polymer matrices without inducing aggregation. The incorporation of these nanoparticles not only enhanced the crystallinity to some extent but also improved tensile strength and elasticity and did not hinder the reformation after physical damage. Consequently, the hydrogel demonstrated accelerated self-healing time (under high temperature) without sacrificing its overall toughness, presenting a versatile material platform with great potential for applications in biomedical devices, tissue engineering scaffolds, and other advanced functional systems.

## 2. Materials and Methods

### 2.1. Materials

PVA (Mowiol^®^ 28-99, Mw = ~145,000 g/mol, degree of hydrolysis > 99%) was obtained from Sigma-Aldrich (Darmstadt, Germany). PVA (Mw = ~88,100 g/mol, degree of hydrolysis ≥ 98.5%) and PVA (Mw = ~88,100 g/mol, degree of hydrolysis 80%) were purchased from Nacalai Tesque (Kyoto, Japan). Suspensions of Si nanoparticles (average diameters of 12, 85, and 200 nm) were obtained from Nissan Chemical Industries Ltd. (Tokyo, Japan).

### 2.2. Preparation of PVA Hydrogels

To identify the most suitable PVA material for rapid self-healing, the following grades were compared:

- P1 = Mw ~88,100 g/mol, degree of hydrolysis ≥98.5%;
- P2 = Mw ~88,100 g/mol, degree of hydrolysis 80%;
- P3 = Mw ~145,000 g/mol, degree of hydrolysis >99%.

Hydrogels were fabricated by dissolving PVA (15 g) in water (85 mL) and heating the blend at 95 °C under continuous stirring for 4 h. A 5 wt% Si nanoparticle solution was then added to achieve a final concentration of 0.05 wt% relative to the mixture. The prepared solution was cast into a square styrene mould, frozen at −15 °C for 24 h, and thawed at RT for 4 h. Freeze/thaw (F/T) cycles were repeated several times to compare polymer crystallization behaviour with Si-induced crystal growth.

### 2.3. Characterization Methods

#### 2.3.1. Mechanical Strength

Tensile stress–strain measurements were performed using a universal testing machine (Shimadzu EZ-LX, 100 N, Tokyo, Japan) at a strain rate of 50 mm/min. Hydrogel specimens were cut into dumbbell shapes of 17 mm height, 2 mm width, and 3 mm thickness.

#### 2.3.2. Crystallization Properties

The crystallization behaviour of hydrogels was analyzed using X-ray diffraction (XRD; Smart Lab, Rigaku, Tokyo, Japan). The hydrogels were first frozen in liquid nitrogen and subsequently freeze-dried under vacuum at −50 °C for 48 h using a freeze dryer (FRD-mini, IWAKI, Asahi Techno Glass, Tokyo, Japan) to ensure complete solvent sublimation. XRD measurements were performed over a 2θ range of 2–50°.

The degree of crystallinity (*Xc*) was calculated as follows:

(1) $$Xc=\left(\frac{Ac}{Ac+Aa}\right)\times 100,$$

where *A*c is the area under the crystalline peaks and *A*c + *A*a is the total area of the amorphous and crystalline peaks.

The crystal size was calculated using the Debye–Scherrer equation:

(2) $$D=\frac{K\lambda }{\beta cos\theta }\;\;,$$

where D is the crystal size (nm), K = 0.9 is the Scherrer constant, λ  = 0.15406 nm (wavelength of X-ray sources), β is the full width at half maximum (FWHM) of the diffraction peak in radians, and θ is the peak position in radians.

#### 2.3.3. Swelling Behaviour

Hydrogel samples (5 mm thick) were cut into pieces measuring 15 mm × 10 mm. The samples were immersed in deionized water at 20 °C, and at specified time intervals, the swollen samples were removed and weighed. To ensure accuracy, each sample was measured three times. The degree of swelling of hydrogels was calculated using the following equation:

(3) $$Degree\;of\;swelling\;(\%)=\frac{\left(W_{t}-W_{d}\right)}{W_{d}}\times 100,$$

where *W*_*t*_ represents the weight of the swollen (rehydrated) hydrogel at time *t*, while *W*_*d*_ denotes the weight of the freeze-dried sample.

## 3. Results and Discussion

### 3.1. Hydrogel Synthesis

The PVA hydrogel was prepared via heating, cooling, and F/T. An aqueous PVA solution was mixed with 0.05 wt% colloidal Si nanoparticles, heated at 95 °C for 4 h under mechanical stirring, and cooled to RT (25 °C). Finally, the solution was frozen at −15 °C for 24 h and thawed at room temperature for 4 h. Turturro et al. found that minimal Si addition (<1 part per 100 parts PET) greatly enhanced poly (ethylene terephthalate) PET crystallization, while higher concentrations slowed it below that of the unmodified polymer [17]. Thus, a low silica content was chosen to promote crystal growth in PVA.

Aqueous PVA solutions (H1, H2, and H3) were prepared using different grades of PVA (P1, P2, and P3) to identify the most suitable material exhibiting self-healing ability in different temperature conditions. After selecting the suitable PVA grade, hydrogels were subsequently fabricated with and without silica nanoparticles for comparative analysis.

Multiple F/T cycles were conducted to evaluate the influence of Si loading on crystallinity relative to pure PVA hydrogels.

As shown in Table 1, the gelation ability of aqueous solutions of PVA, differing in molecular weight and degree of hydrolysis, was first evaluated. H1 and H3 formed stable hydrogels (turbid white) within one F/T cycle, indicating that medium- and high-molecular-weight PVA with higher degrees of hydrolysis are more suitable for hydrogel formation. H2 did not form a hydrogel; it resulted in a viscous solution. A higher degree of hydrolysis increases the substitution of acetate groups with hydroxyl (–OH) groups, thereby enhancing intra- and intermolecular hydrogen bonding. This improved hydrogen bonding promotes PVA chain crystallization during F/T processing [18,19]. Based on these findings, only H1 and H3 were selected for further investigation.

### 3.2. Self-Healing Mechanism

**Figure 1:**
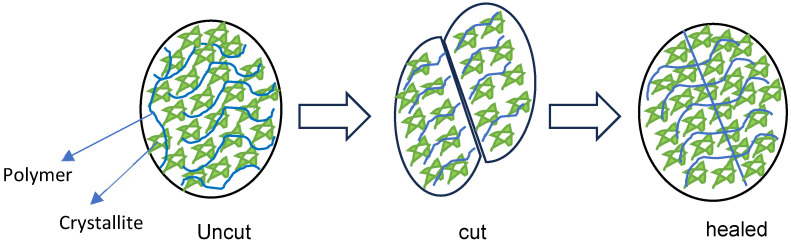
Hydrogel healing process.

**Figure 2:**
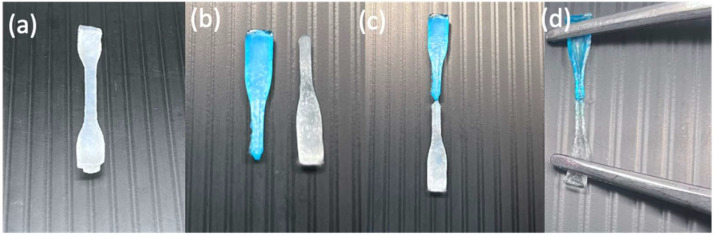
Digital images of an H1 hydrogel sample (turbid white) during self-healing at RT: hydrogel sample (**a**) before cutting, (**b**) after being cut in the middle and separated in two (one colored with methylene blue and the other kept the same), (**c**) after being reattached for self-healing, and (**d**) after self-healing while being stretched.

The self-healing behaviour of hydrogels is generally governed by dynamic and reversible interactions such as hydrogen bonding, ionic interactions, host–guest chemistry, or dynamic covalent bonds (e.g., Schiff base or disulfide bonds). When the hydrogel is damaged, these reversible bonds reform across the fracture interface, restoring structural integrity [20,21,22]. As shown in Figure 1 and Figure 2, the hydrogel was cut into two equal portions, reattached physically, and then underwent self-healing at RT. It was observed that hydrogels composed of shorter polymer chains (medium molecular weight) exhibit greater chain mobility in achieving self-healing faster, whereas hydrogels formed from longer polymer chains (higher molecular weight) were slower and required more time to achieve adequate self-healing. Kamiyama et al. demonstrated that lower-molecular-weight poly (methyl methacrylate) (PMMA)/1-ethyl-3-methylimidazolium bis (trifluoromethyl sulfonyl) imide ([C_2_mIm][TFSI]) gels healed faster due to enhanced chain mobility, but their mechanical properties were greatly compromised [23].

### 3.3. PVA Hydrogel Self-Healing Conditions

The intrinsic self-healing performance of native PVA hydrogels was systematically investigated in Table 2, under both ambient and thermally stimulated. The recovery behaviour of the hydrogel network was first examined at RT (25 °C) for 1 h. Subsequently, additional experiments were performed at 40, 50, and 60 °C, each maintained for 1 h, to assess the influence of thermal activation on the healing process. Among the prepared hydrogels, sample H1 from PVA polymer P1 grade consistently exhibited self-healing across all tested temperatures, indicating its superior dynamic network rearrangement compared to H3. Based on this promising outcome, H1 was selected as the representative hydrogel system for subsequent in-depth investigations. In the next stage, emphasis was placed on improving its mechanical robustness through the incorporation of Si nanoparticles, which were anticipated to act as effective nanofillers and crystal-regulating agents, thereby enhancing the overall structural stability of the hydrogel.

### 3.4. Mechanical Properties of Hydrogels

#### 3.4.1. Self-Healing of PVA Hydrogels (H1) Without Si at Room Temperature

**Figure 3:**
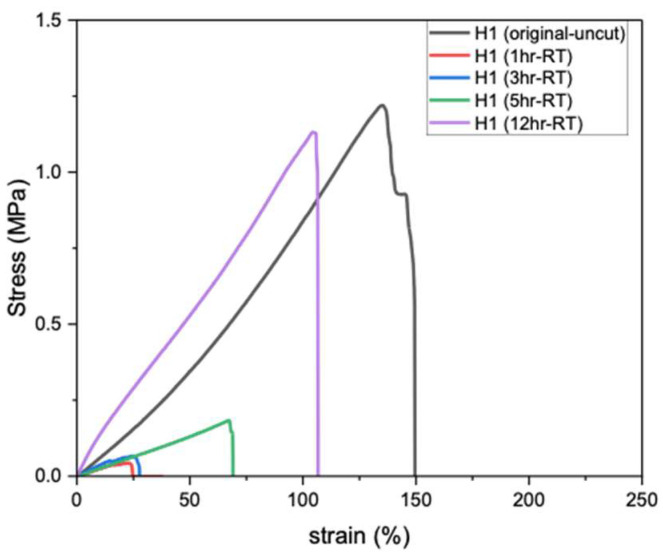
Tensile strength of H1 hydrogels (15 g) after self-healing at RT.

Self-healing of hydrogels at RT (20–25 °C) is particularly important, as it enables material recovery from mechanical damage under ambient conditions without requiring heat, light, or chemical triggers. Figure 3 shows the tensile strength of PVA hydrogels after different healing durations; the original H1 (uncut) exhibits the highest tensile strength (1.21 MPa), whereas after cutting and rejoining, the hydrogel demonstrates very weak recovery after 1 h of self-healing at RT. Extending the healing period to 3, 5, and 12 h gradually increased the tensile strength, reaching a maximum of 94% of the original uncut value after 12 h. Therefore, prolonged healing time (12 h) at RT is required for H1 hydrogels to achieve autonomous self-healing and restore near-original strength [13].

#### 3.4.2. Self-Healing of H1 Hydrogels Without Si Under Thermal Stimulation

**Figure 4:**
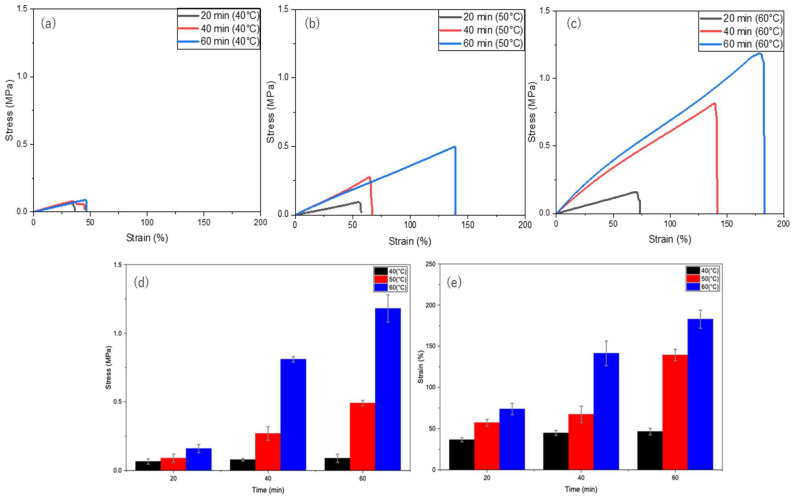
Self-healing of H1 hydrogels (15 g) under thermal stimulation at different temperatures and durations: (**a**) 40 °C, (**b**) 50 °C, (**c**) 60 °C; (**d**) time versus stress at different temperatures (40, 50, and 60 °C); and (**e**) time versus strain at different temperatures (40, 50, and 60 °C).

To achieve rapid self-healing, hydrogel samples were heated at 40, 50, and 60 °C for 20, 40, and 60 min. As shown in Figure 4, the self-healing efficiency gradually increases with both time and temperature. At 40 °C (Figure 4a), the hydrogel reached a maximum of 6% of the original uncut strength, whereas at 50 °C (Figure 4b), recovery improved over time to 33% of the original uncut strength. Finally, the hydrogel achieved the highest recovery of 96% of its original uncut strength at 60 °C (Figure 4c) for 60 min. Therefore, the enhanced mobility of the PVA polymer chains at higher temperatures (60 °C) promotes self-healing compared with that at slightly lower temperatures (40 and 50 °C). Furthermore, heating the H1 hydrogel for 1 h significantly shortened the self-healing time (previously achieved 12 h at RT), with 60 °C for 60 min being the optimal conditions.

#### 3.4.3. Self-Healing of H1 Hydrogels Containing Si Nanoparticles

**Figure 5:**
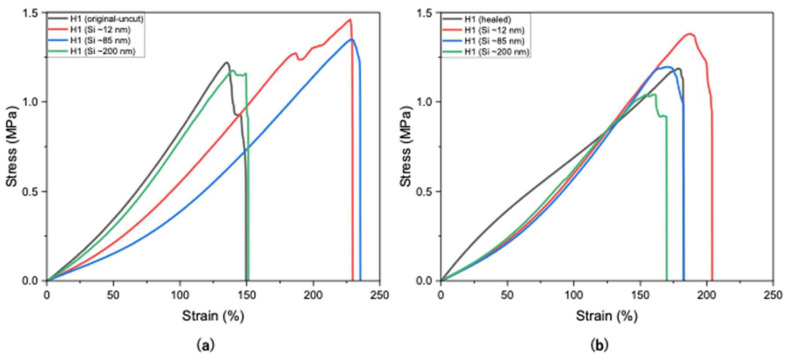
Tensile strengths of H1 hydrogels (15 g) without and with Si (0.05 wt%) nanoparticles (**a**) before cutting and (**b**) after self-healing at optimal conditions.

Si nanoparticles enhance hydrogel properties by significantly improving their mechanical strength, elasticity, and durability. The size of Si nanoparticles plays a critical role in determining their effect on the properties of hydrogels, with smaller particle sizes (~10 to ~30 nm) having a higher surface area-to-volume ratio, thus providing more active sites for interaction with polymer chains. This results in stronger reinforcement, better dispersion, and more effective modification of hydrogel properties. As shown in Figure 5a, H1 hydrogels containing smaller Si particles (~12 nm) exhibited higher tensile strength (1.45 MPa), whereas larger particles (~85 nm and ~200 nm) resulted in reduced strengths of 1.34 MPa and 1.17 MPa, respectively, compared to the unloaded H1 hydrogel (1.21 MPa).

**Figure 6:**
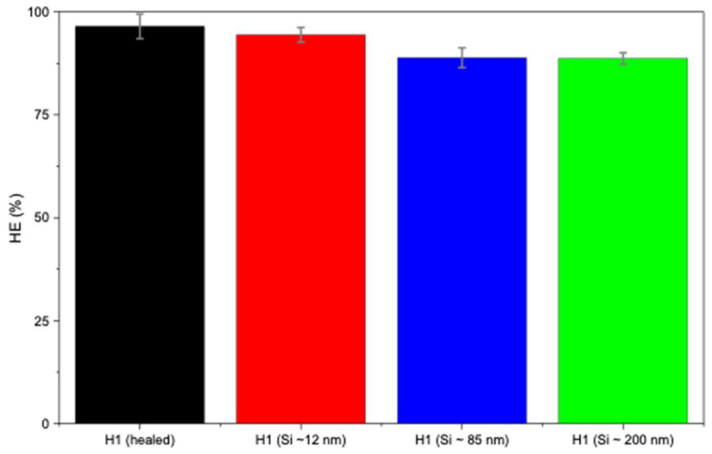
Healing efficiency of H1 hydrogels (15 g) without and with Si (0.05 wt%) nanoparticles.

In Figure 5b, H1 hydrogels loaded with larger Si particles (~85 and ~200 nm) exhibited lower self-healing compared to smaller sizes (~12 nm). This may be attributed to the larger particles not fitting as easily within the polymer matrix, thus leading to weaker nucleation and possibly network disruption. Figure 5b presents the self-healing of H1 hydrogels loaded with Si of various sizes, indicating that Si nanoparticles did not hinder the occurrence of the self-healing mechanism. Smaller Si particles (~12 nm) demonstrated little to negligible hindrance on the self-healing mechanism (94%), whereas sizes of ~85 nm and ~200 nm sharply decreased the healing efficiency (88% and 86%, respectively; Figure 6). Overall, Si nanoparticles significantly improved mechanical strength compared with unloaded H1 hydrogels with the same initial polymer weight (15 g) while not hindering the intrinsic self-healing mechanism.

### 3.5. XRD Results

XRD is a valuable tool for analyzing the degree of crystallinity of polymers by distinguishing between crystalline and amorphous regions, thus providing insights into their mechanical properties [24]. XRD provides information on crystallite size, which is essential for understanding anisotropic behaviour. Additionally, XRD results reveal how additives or fillers such as Si nanoparticles affect the formation and stability of crystalline domains.

**Figure 7:**
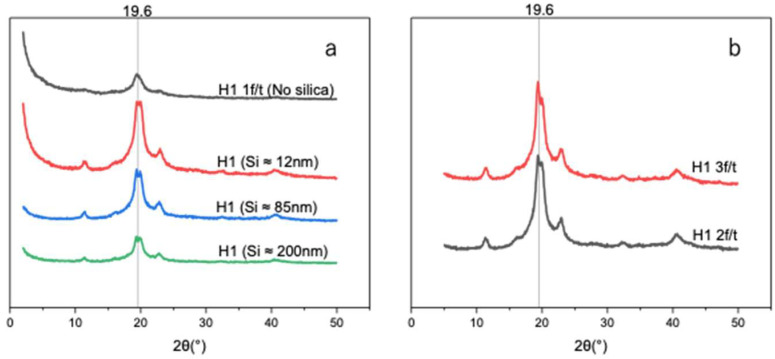
(**a**) X-ray diffraction patterns of unloaded H1 (15 g) with 1 F/T and H1 (15 g) loaded with different sizes of Si (0.05 wt%) nanoparticles. (**b**) X-ray diffraction patterns of self-nucleated H1 (2 F/T and H1 3 F/T).

As shown in Figure 7a, the introduction of Si nanoparticles increased the hydrogel crystallinity degree, with H1 hydrogels containing smaller Si particles (~12 nm) exhibiting a degree of crystallinity of 29.0% compared to 14.3% for H1 (1 F/T) without Si (Table 3). Larger particles (~85 and ~200 nm), which have lower surface area-to-volume ratios resulting in reduced nucleation sites, yielded lower degrees of crystallinity—27.5% and 19.5%, respectively. Such a finding can be attributed to the fact that smaller Si particles (~12 nm), having abundant active sites, promote nucleation and facilitate PVA chain alignment and crystallization. The enhancement of the degree of crystallinity is in correlation with the improvement in mechanical properties upon the introduction of smaller particles (~12 nm), followed by a sharp decrease as the nanoparticle size increases (~85 and ~200 nm).

Numerous studies reported that PVA-based hydrogel could self-nucleate without any additional nucleating agent. Thomas et al. reported that self-nucleation experiments reveal how melt history and memory govern PVA crystallization, with residual crystal fragments or aligned chain regions serving as self-seeded nuclei that enhance nucleation density and raise crystallization onset upon cooling [25].

The distinction in crystallization was assessed by determining the degree of crystallinity of Si-nucleated and self-nucleated H1 hydrogels. In Figure 7a,b, we investigated and compared the degree of crystallinity and average crystallite size. Self-nucleated H1 was triggered under a repeated F/T process. Figure 7b shows that self-nucleated H1 hydrogels under two and three F/T cycles exhibit enhanced XRD intensities with crystallinity values of 39.1% and 44.3% (Table 4), respectively. However, the Si-nucleated H1 hydrogel demonstrated an increased XRD pattern with a smaller size (~12 nm) of Si, while sharply decreasing as the Si size became bigger. Therefore, the Si-nucleated H1 hydrogel is size dependent. In terms of the average crystal size (Table 3 and Table 4), both approaches exhibited consistent size (4 to ~6 nm) across samples, at peak 19.6°.

In summary, both approaches (Si-nucleated and self-nucleating) lead to an increase in crystallinity degree that could be translated to higher mechanical strength. However, it has been reported by Taylor et al. that while F/T cycling enhances the mechanical properties of PVA hydrogels, increasing the cycles from zero to three drastically reduced the self-healing efficiency from 68% to about 10% [26]. Therefore, the self-nucleated H1 hydrogel approach promises good mechanical strength but compromises the self-healing properties.

In that regard, H1 hydrogel nucleated by Si nanoparticles of smaller size (12 nm) remains the most effective method to achieve enhanced mechanical strength and excellent self-healing properties.

### 3.6. Effect of Si on Hydrogel Swelling Performance

**Figure 8:**
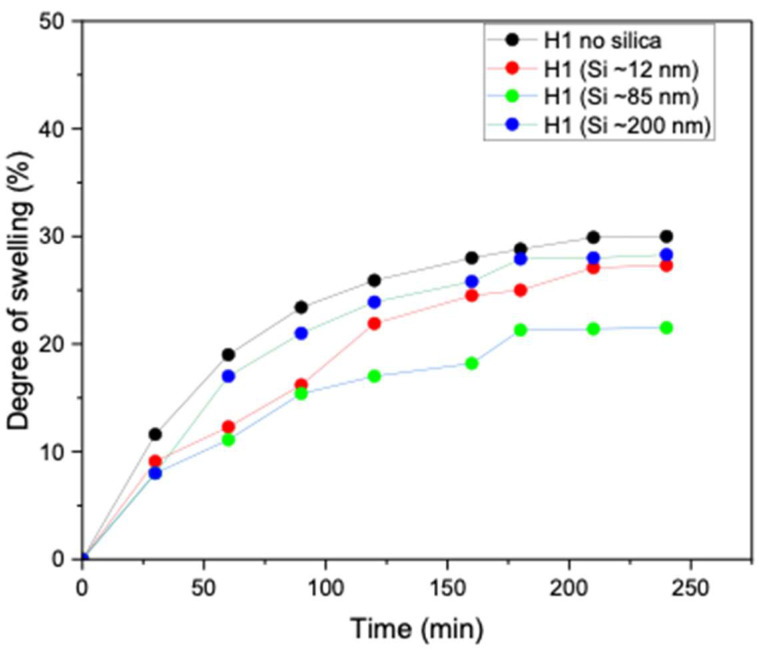
Degree of swelling of unloaded H1 (15 g) 1 F/T and H1 (15 g) loaded with Si (0.05 wt%) nanoparticles (~12, ~85, and ~200 nm).

Swelling behaviour is a critical property of hydrogels, as it controls water uptake, dimensional stability, and responsiveness to external stimuli. The degree of swelling directly influences key characteristics such as mechanical strength, porosity, and the overall functional performance of hydrogels in practical applications. As shown in Figure 8, the unloaded H1 hydrogel (no silica) exhibited high water absorption capacity (more than 30%). However, upon incorporation of Si nanoparticles, a clear size-dependent effect was observed. Specifically, the introduction of smaller and medium nanoparticles (~12 and ~85 nm) markedly reduced water uptake, which is attributable to the denser hydrogel network formed owing to enhanced crystallinity compared to the unloaded H1 hydrogel. In contrast, H1 hydrogels loaded with larger Si nanoparticles (~200 nm) exhibited lower crosslinking density, thereby maintaining a high degree of swelling, similar to that of the unloaded hydrogel. These findings demonstrate that nanoparticle size, degree of crystallinity, and crosslinking density govern the swelling performance of H1 hydrogels. The studies are in line with Zaragoza et al., who showed that incorporating Si nanoparticles into poly(acrylamide) hydrogels significantly reduced swelling due to nanoparticle-mediated pseudo-crosslinking [27].

## 4. Conclusions

In this study, we established a straightforward strategy to achieve mechanical reinforcement in PVA-based hydrogels while maintaining their self-healing properties. Leveraging intrinsic dynamic interactions within the polymer network, the system remains environmentally friendly and biocompatible while retaining resilience under mechanical stress. The application of a mild thermal stimulus (60 °C) shortened the healing period from 12 h at RT to just 60 min, confirming the role of thermal activation in accelerating network reformation. In parallel, the incorporation of ultra-small Si nanoparticles (12 nm) further enhanced mechanical strength by acting as an efficient nucleating agent while preserving high self-healing efficiency. In contrast to the findings of Lee et al. [9], who reported an increase in the degree of crystallinity at 0.5 wt% Si nanoparticle loading in the PVA matrix, the present study achieved a comparable nucleation effect at a substantially lower concentration of only 0.05 wt%, demonstrating the strong nucleating efficiency of the incorporated Si nanoparticles. Collectively, these findings demonstrate a versatile platform for multifunctional hydrogels with broad potential in soft robotics and wound dressing, where durability, adaptability, and autonomous repair are essential.

## Figures and Tables

**Table 1:** Preparation conditions of aqueous PVA solutions and their gelation ability.

Aqueous PVA Solution	PVA		F/T Cycle (Times)	Gel Formation ^a^
	Grade	Weight (g)		
H1	P1	15	1	G
H2	P2	15	1	VS
H3	P3	15	1	G

**^a^** G: gelation; VS: viscous solution.

**Table 2:** Hydrogel self-healing temperature conditions tested.

Samples	Temperature			
	RT (25 °C)	40 °C	50 °C	60 °C
H1	SHF	SHF	SHF	SHF
H3	NSHF	NSHF	SHF	SHF

SHF, self-healing formed; NSHF, no self-healing formed.

**Table 3:** Crystallinity degree of H1 hydrogels with and without Si nanoparticles and their average crystal size at 19.6 peak position.

PVA Samples	Crystallinity %	Average Crystal Size (nm)
H1 1 F/T (no silica)	14.3 ±1.3	5.9
H1 (Si ~12 nm)	29.0 ±0.9	4.9
H1 (Si ~85 nm)	27.5 ±1.0	4.2
H1 (Si ~200 nm)	19.5 ±1.6	4.1

**Table 4:** Crystallinity of H1 hydrogels without Si, induced in multiple F/T cycles, and average crystal size at 19.6 peak position.

PVA Samples	Crystallinity (%)	Average Crystal Size (nm)
H1 2 F/T	39.1 ± 1.1	4.1
H1 3 F/T	44.3 ± 1.5	4.1

## Data Availability

The data presented in this study are openly available in the article.

## Abbreviations

The following abbreviations are used in this manuscript:

RT	Room temperature
PVA	Polyvinyl alcohol
F/T	Freeze/thaw
Si	Silica

## References

[1] Kumar B., Sauraj, Negi Y.S. (2019). To Investigate the Effect of Ester-Linkage on the Properties of Polyvinyl Alcohol/Carboxymethyl Cellulose Based Hydrogel. Mater. Lett..

[2] Wang C., Du Y., Chen B., Chen S., Wang Y. (2019). A Novel Highly Stretchable, Adhesive and Self-Healing Silk Fibroin Powder-Based Hydrogel Containing Dual-Network Structure. Mater. Lett..

[3] Wang Z., Tao F., Pan Q. (2016). A Self-Healable Polyvinyl Alcohol-Based Hydrogel Electrolyte for Smart Electrochemical Capacitors. J. Mater. Chem. A Mater..

[4] Meng H., Xiao P., Gu J., Wen X., Xu J., Zhao C., Zhang J., Chen T. (2014). Self-Healable Macro-/Microscopic Shape Memory Hydrogels Based on Supramolecular Interactions. Chem. Commun..

[5] Li J., Geng L., Wang G., Chu H., Wei H. (2017). Self-Healable Gels for Use in Wearable Devices. Chem. Mater..

[6] Ashraful Alam M., Takafuji M., Ihara H. (2013). Thermosensitive Hybrid Hydrogels with Silica Nanoparticle-Cross-Linked Polymer Networks. J. Colloid. Interface Sci..

[7] Wang K., Cheng W., Ding Z., Xu G., Zheng X., Li M., Lu G., Lu Q. (2021). Injectable Silk/Hydroxyapatite Nanocomposite Hydrogels with Vascularization Capacity for Bone Regeneration. J. Mater. Sci. Technol..

[8] Yang J., Zhao J. (2014). Preparation and Mechanical Properties of Silica Nanoparticles Reinforced Composite Hydrogels. Mater. Lett..

[9] Lee J., Jin Lee K., Jang J. (2008). Effect of Silica Nanofillers on Isothermal Crystallization of Poly(Vinyl Alcohol): In-Situ ATR-FTIR Study. Polym. Test..

[10] Chen J., Lv Q., Wu D., Yao X., Wang J., Li Z. (2016). Nucleation of a Thermoplastic Polyester Elastomer Controlled by Silica Nanoparticles. Ind. Eng. Chem. Res..

[11] Annaka M., Mortensen K., Matsuura T., Ito M., Nochioka K., Ogata N. (2012). Organic-Inorganic Nanocomposite Gels as an in Situ Gelation Biomaterial for Injectable Accommodative Intraocular Lens. Soft Matter.

[12] Yang J., Han C.R., Duan J.F., Xu F., Sun R.C. (2013). Insitu Grafting Silica Nanoparticles Reinforced Nanocomposite Hydrogels. Nanoscale.

[13] Zhang H., Xia H., Zhao Y. (2012). Poly(Vinyl Alcohol) Hydrogel Can Autonomously Self-Heal. ACS Macro Lett..

[14] Zengin A., Castro J.P.O., Habibovic P., Van Rijt S.H. (2021). Injectable, Self-Healing Mesoporous Silica Nanocomposite Hydrogels with Improved Mechanical Properties. Nanoscale.

[15] Huang X., Zhou X., Zhou H., Zhong Y., Luo H., Zhang F. (2021). A High-Strength Self-Healing Nano-Silica Hydrogel with Anisotropic Differential Conductivity. Nano Res..

[16] Zheng J., Xiao P., Liu W., Zhang J., Huang Y., Chen T. (2016). Mechanical Robust and Self-Healable Supramolecular Hydrogel. Macromol. Rapid Commun..

[17] Turturro G., Brown G.R., St-Pierre L.E. (1984). Effect of Silica Nucleants on the Rates of Crystallization of Poly(Ethylene Terephthalate). Polymer.

[18] Holloway J.L., Lowman A.M., Palmese G.R. (2013). The Role of Crystallization and Phase Separation in the Formation of Physically Cross-Linked PVA Hydrogels. Soft Matter.

[19] Zhang A., Huang H., Shen J., Feng X., Duan L., Wang J., Zhang X. (2025). A Synergistic “Pre-Crosslinking-Freeze Thawing-Salting out-Coordination” Tactic to Design Antimicrobial and Highly Conductive PVA/PEI Hydrogels with Excellent Mechanical Performance. Chem. Eng. J..

[20] Shao J., Zhang Z., Zhao S., Wang S., Guo Z., Xie H., Hu Y. (2019). Self-Healing Hydrogel of Poly (Vinyl Alcohol)/Agarose with Robust Mechanical Property. Starch.

[21] Samadi N., Sabzi M., Babaahmadi M. (2018). Self-Healing and Tough Hydrogels with Physically Cross-Linked Triple Networks Based on Agar/PVA/Graphene. Int. J. Biol. Macromol..

[22] Xu K., Wang Y., Zhang B., Zhang C., Liu T. (2021). Stretchable and Self-Healing Polyvinyl Alcohol/Cellulose Nanofiber Nanocomposite Hydrogels for Strain Sensors with High Sensitivity and Linearity. Compos. Commun..

[23] Kamiyama Y., Tamate R., Hiroi T., Samitsu S., Fujii K., Ueki T. (2022). Highly Stretchable and Self-Healable Polymer Gels from Physical Entanglements of Ultrahigh-Molecular Weight Polymers. Sci. Adv..

[24] Mahamoud M.M., Ketema T.M., Kuwahara Y., Takafuji M. (2024). Enhancement of Mechanical Properties of Benign Polyvinyl Alcohol/Agar Hydrogel by Crosslinking Tannic Acid and Applying Multiple Freeze/Thaw Cycles. Gels.

[25] Thomas D., Cebe P. (2017). Self-Nucleation and Crystallization of Polyvinyl Alcohol. J. Therm. Anal. Calorim..

[26] Taylor D.L., In Het Panhuis M. (2016). Self-Healing Hydrogels. Adv. Mater..

[27] Zaragoza J., Babhadiashar N., O’Brien V., Chang A., Blanco M., Zabalegui A., Lee H., Asuri P. (2015). Experimental Investigation of Mechanical and Thermal Properties of Silica Nanoparticle-Reinforced Poly(Acrylamide) Nanocomposite Hydrogels. PLoS ONE.

